# Reconstruction of the yeast protein-protein interaction network involved in nutrient sensing and global metabolic regulation

**DOI:** 10.1186/1752-0509-4-68

**Published:** 2010-05-25

**Authors:** Subir K Nandy, Paula Jouhten, Jens Nielsen

**Affiliations:** 1 Systems Biology Group, Department of Chemical and Biological Engineering, Chalmers University of Technology, Kemivägen 10, SE-412 96, Gothenburg, Sweden

## Abstract

**Background:**

Several protein-protein interaction studies have been performed for the yeast *Saccharomyces cerevisiae *using different high-throughput experimental techniques. All these results are collected in the BioGRID database and the SGD database provide detailed annotation of the different proteins. Despite the value of BioGRID for studying protein-protein interactions, there is a need for manual curation of these interactions in order to remove false positives.

**Results:**

Here we describe an annotated reconstruction of the protein-protein interactions around four key nutrient-sensing and metabolic regulatory signal transduction pathways (STP) operating in *Saccharomyces cerevisiae*. The reconstructed STP network includes a full protein-protein interaction network including the key nodes Snf1, Tor1, Hog1 and Pka1. The network includes a total of 623 structural open reading frames (ORFs) and 779 protein-protein interactions. A number of proteins were identified having interactions with more than one of the protein kinases. The fully reconstructed interaction network includes all the information available in separate databases for all the proteins included in the network (nodes) and for all the interactions between them (edges). The annotated information is readily available utilizing the functionalities of network modelling tools such as Cytoscape and CellDesigner.

**Conclusions:**

The reported fully annotated interaction model serves as a platform for integrated systems biology studies of nutrient sensing and regulation in *S. cerevisiae*. Furthermore, we propose this annotated reconstruction as a first step towards generation of an extensive annotated protein-protein interaction network of signal transduction and metabolic regulation in this yeast.

## Background

The development of high-throughput analytical methods for genes and gene products and the wealth of information obtained in recent years combined with extensive annotation allows for a genome-wide view on the *Saccharomyces cerevisiae *proteome. In systems biology studies of signal transduction pathways, the natural first step to model the dynamics operation of these pathway are to identify the proteins acting in the studied pathway and the interactions between them. Therefore there is an increasing interest in identification of all protein-protein interactions in the model organism *S. cerevisiae *[1,2], and in recent studies the protein-protein interactions for single protein kinases were mapped [3,4].

There have been many attempts to reconstruct signal transduction pathways (STPs) [5], and extensive databases are available, e.g. BIOGRID, SGD, containing information on the components of different STPs. There is also an increasing amount of data on how proteins assemble in cells. The analysis of all this data has allowed for identification of new pathways and also refined our models for previously known pathways [4], and using protein chips more than 4000 phosphorylation events including 1325 different proteins have been identified by Snyder et. al., 2005.

The full datasets of protein-protein interaction used in our study were available from different sources such as the literature, large scale microarray experiments and whole genome two hybrid screenings. Whole protein-protein interaction networks allows for inferring protein networks that are involved in the same cellular processes [6], and through comparison with published data the most likely topologies of specific pathways can be identified and this can be used to design further experiments that can test the different predictions.

In this study we present the first step towards an integrated reconstruction of key signal transduction pathways in *S. cerevisiae*. Our focus is on the interactions of the protein kinases Snf1, Tor1, Hog1 and Pka1, as these protein kinases play a central role in regulation of nutrient uptake, energy, carbon and nitrogen metabolisms. These four protein kinases are all involved in the regulation of energy homeostasis in the cell. Snf1 is one of the main regulators of the diauxic shift from fermentative to respirative metabolic state in *S. cerevisiae *[7] and its mammalian counterpart, AMPK, is a metabolic regulator involved in activation of catabolic processes such as β-oxidation and repression of energy consuming reactions such as lipid biosynthesis, and hereby it plays a central role in metabolic disorders such as diabetes and the metabolic syndrome [8]. TOR controls cell growth in response to nutrient availability and stress and it exists in *S. cerevisiae *in two structurally and functionally distinct protein complexes termed TorC1 and TorC2 [9]. Global nutrient-sensing signal transduction cascades like TOR and RAS activate Pka1 in response to glucose availability [10]. Protein kinase A, or Pka1, is a key player in regulation of carbon metabolism. When the cAMP concentration increases and binds to the regulatory subunits of protein kinas A, the subunits dissociate from the protein complex and the kinase is activated [11]. Stress resistance of *S. cerevisiae *is to a large extent dependent on the protein kinase Hog1, which among other things regulate the glycerol production in response to hyper osmotic conditions [12]. Since these four protein kinases are key players in regulation of energy metabolism and highly conserved among eukaryotes [3], they are also of relevance in understanding of metabolic diseases such as the metabolic syndrome related diseases, such as arteriosclerosis, diabetes type II and hypertension. Therefore, understanding the integrated function of these protein kinases is of great importance in development of effective therapies for metabolic diseases. The four protein kinases are also considered to be crucial elements of transcriptional, metabolic and developmental regulation in response to stress [13-15]. Inactivation of Hog1 has for example been observed to significantly attenuate the transcriptional response to osmotic stresses [16].

Reconstruction of protein-protein interactions are important for understanding cellular networks [17], and based on recent development of new high throughput technologies PPIs have accumulated rapidly [18]. There are several methods available to find PPIs such as *Yeast Two Hybrid *[19,20], *Tandem Affinity Purification *and computational method like *Phylogenic profile*, *Correlated Domain Signature Method *and *Integrative methods *[21]. Most of these approaches cover only a sub-set of possible interactions. Wang et. al., 2009 tried to integrate protein-protein features from multiple data sources [21]. Besides the reconstruction of PPIs there have also been developed different methods for analysis of these networks [22,23], such as residue spatial sequence profile and evolution rate [24], structural information [25], and residue conservation scores [26].

Here the reconstructed network is presented in a platform that will allow continued annotation, e.g. in a community based effort in analogy with what has recently been done for genome-scale metabolic models of yeast [27]. Databases like SGD, BioGRID and several papers describe the function and interaction of the individual kinases, but there has so far not been an made an integrated interaction networks of these four key protein kinases that unite all key nutrient and energy sensing STPs in *S. cerevisiae*. The aim of our reconstruction is to present the first integrated network reconstruction of how these four key protein kinases interact, with annotations of the different interactions according to the current knowledge, and further evaluate the role of this interaction network in global metabolic regulation. We specifically wanted to study how these four protein kinase function in concert and we therefore only considered direct neighbours of these protein kinases. The reconstructed protein-protein network is analyzed in terms of its connectivity, and the network is used as a framework for analysis of transcriptome data of *S. cerevisiae *grown at different environmental conditions expected to affect the function of these key STP.

## Results

### Annotations for protein-protein interactions

**Figure 1:**
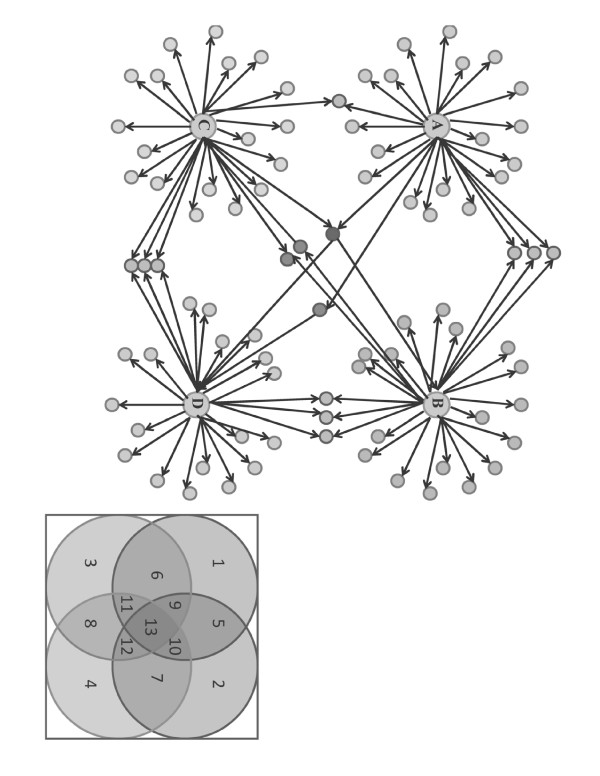
**The process of the reconstruction of the key signal transduction pathways embedded in the global protein-protein interaction network of *S. cerevisiae***. From different types of available information such as different biochemical pathway databases, different textbooks, recent publications and reviews and genome annotations, a physical reconstruction of a protein-protein interaction network was designed and annotated so that each protein and their interactions include all information. The pathway map picture was taken from the KEGG database (www.genome.ad.jp) and SGD, Biogrid, Cytoscape and CellDesigner logos were taken from their respective websites.

Towards the aim of reconstructing a global protein-protein interaction map for *S. cerevisiae*, we focused on protein interactions with the four key protein kinases Snf1, Tor1, Hog1 and Pka1. These four protein kinases are highly conserved among eukaryotic cells and they play a central role in integrating cellular responses to nutrients and stress, and hereby balancing metabolic functions to ensure proper cell growth and proliferation. Due to their central role in regulating key pathways these protein kinases are also key drug targets for many important diseases e.g. diabetes and cancer. The protein-protein interaction network around these four protein kinases was reconstructed and annotated in CellDesigner using the following steps (see Figure 1).

a. For each of the four key protein kinases the interaction neighbors were identified using different databases and literature information. Each interaction was annotated into the following categories:

(i) Known physical interactions

(ii) Known functional interactions

b. All interactions were annotated with available information as references in the CellDesigner model.

c. The reconstructed interaction network was integrated into a novel visualization mode named the Binary Matrix. The Binary Matrix is a simple representation of the whole interaction network with annotations for each protein and interaction stored in an Excel sheet.

d. Finally the reconstructed sub-networks of each of the protein kinases were combined into one super-network through shared proteins.

Thus, the large-scale protein-protein interaction reconstruction includes four key protein kinases and their neighboring proteins, and with the use of the CellDesigner platform the reconstructed model can easily be represented in SBML. Furthermore, the network is curated with 154 references and it therefore also represents a valuable database on protein-protein interaction.

**Figure 2:**
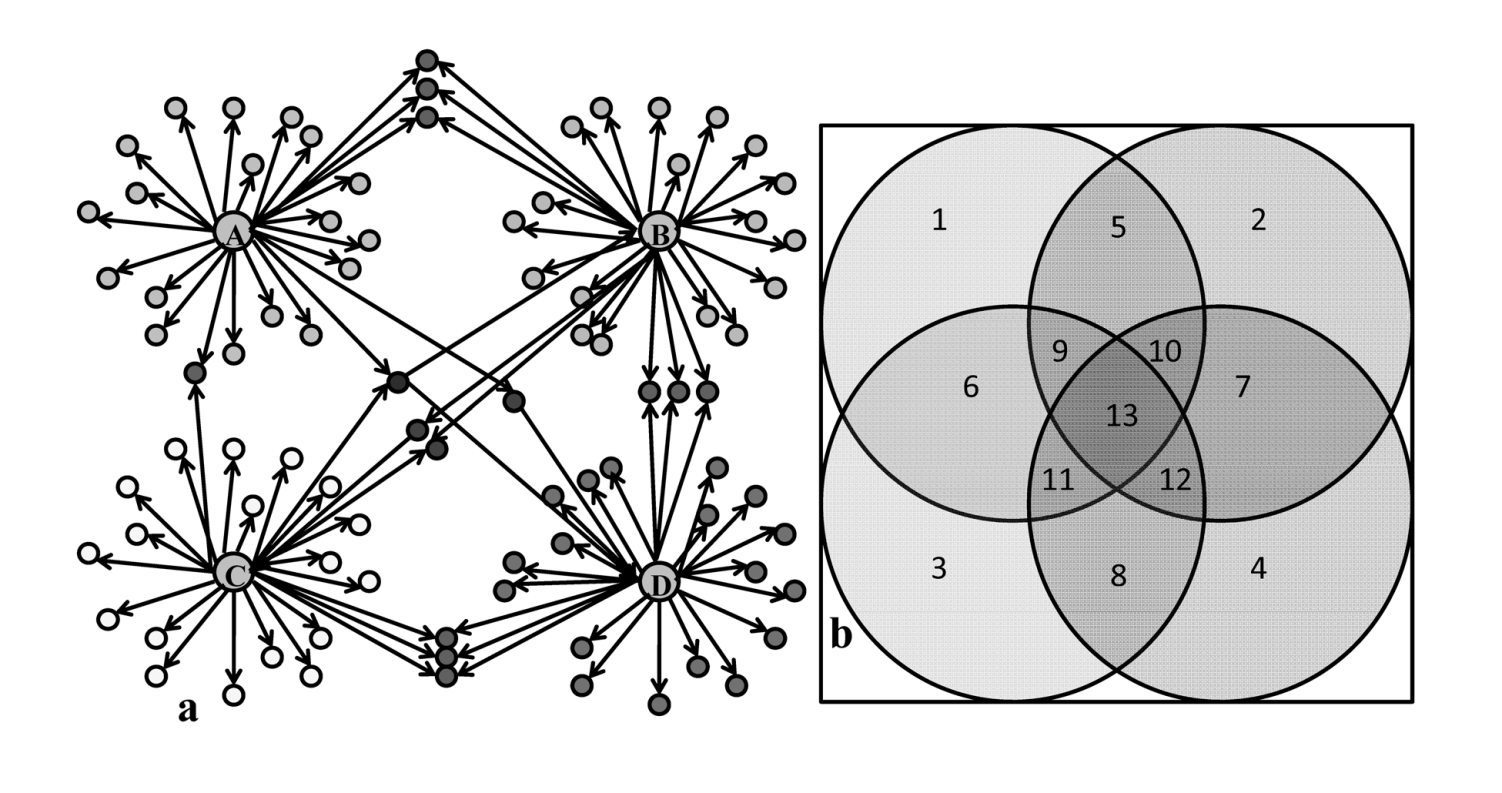
**A global protein-protein interaction network of four key protein kinases was reconstructed and annotated**. The protein-protein interaction map includes four key protein kinases and their neighbouring proteins. (a) The four key protein kinases interact with other proteins: Ash color: direct interaction, Deep Ash color: diagonal interaction and Red color: more than two proteins interact with the same protein. (b) Positions 1, 2, 3, and 4 show the neighbors of the four key proteins; Positions 5, 6, 7, and 8 show the proteins having interactions with any two of the four key proteins; Positions 9, 10, 11 and 12 show the proteins having interactions with any three of the four key proteins; and Position 13 shows the proteins having interactions with all the four key proteins.

The 4 key proteins connect with other proteins in different combinations (see Figure 2a), and these connections can be represented as a Venn diagram that indicates the overlap in interaction around the four protein kinases (Figure 2b). The positions of the proteins in the network are defined by the number 1 to 13 in the Venn diagram. From the reconstructed protein-protein interaction network, the number of neighbors of the four key protein kinases could be identified (see Table 1).

**Table 1:** Schematic representation of the reconstruction of the protein-protein interaction map of the four key protein kinases of *S. cerevisiae*.

	Snf1	Tor1	Pka1	Hog1
**Snf1**	241	0	0	0

**Tor1**	0	129	0	0

**Pka1**	0	0	300	0

**Hog1**	0	0	0	98

### Information on second and third order interactions

**Figure 3:**
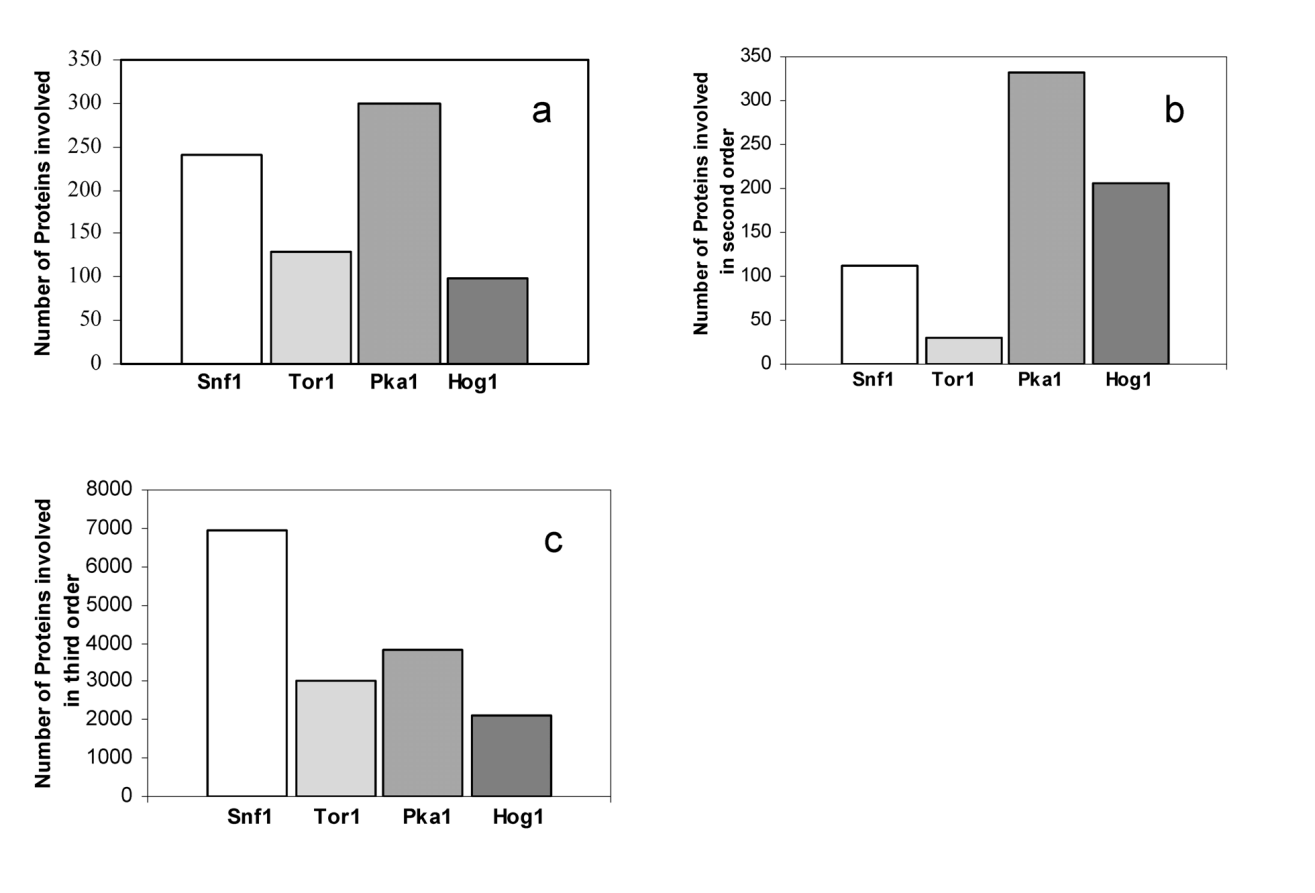
**Number of proteins and ORF's of *S. cerevisiae *included in the protein-protein interaction map with the key protein kinases in (a) first order, (b) second order and (c) third order**. The four key protein kinases are sorted based on metabolism, energy and nutrient sensing metabolism.

From the Venn diagram the numbers of neighboring proteins to the key protein kinases was found and the results are summarized in Figure 3. It is found that Pka1 interacts with most proteins while Snf1 is the second largest hub in the network.

To mathematically represent the structure of the complex protein-protein interaction network, we converted the interactions into a square matrix where all the proteins are listed in both the row and column dimensions and the matrix elements represent interactions between two proteins. If there is an interaction between two proteins the matrix element is set to 1 and if there is no interaction it is set to 0 as a sparse array. This binary representation of protein-protein interactions in square matrix form is an approach equivalent to adjacency matrix in graph theory that enables straightforward analysis of second and third order interactions in the network. Taking a square of the initial Binary matrix returns the second order interactions through one intermediate protein (two edges). Similarly, if a square of the second order matrix is taken, the third order interactions through two intermediate proteins (three edges) are directly obtained. The numbers of intermediate proteins involved in the second and the third order interactions for each protein kinase are shown in Figures 3b and 3c, respectively. Pka1 and Snf1 are found to have the highest number of proteins involved in their second and third order interactions, respectively.

**Figure 4:**
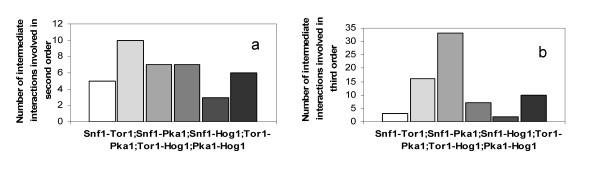
**Number of interactions involved in the protein-protein interaction map for *S. cerevisiae *in the combinations of intermediate protein- protein interactions in (a) second and (b) third order**.

Figure 4 shows the second and third order intermediate interactions obtained from the multiplication of the Binary matrix (see additional file 1 for the binary matrix of proteins used in this study). Here it is assumed that these interactions can be found solely from the upper triangular matrix, meaning that it is assumed that the interactions do not possess directionalities. From the reconstructed protein-protein interaction network, the number of neighbors of the four key protein kinases and their intermediate proteins in second order could also be identified (see Table 2).

**Table 2:** Reconstruction of the second order protein-protein interactions and intermediate proteins involved in the protein-protein interaction map of the four key protein kinases of *S. cerevisiae*.

	Snf1	Tor1	Pka1	Hog1
**Snf1**	112	5	10	7

**Tor1**	0	31	7	3

**Pka1**	0	0	333	6

**Hog1**	0	0	0	206

Figure 4a and 4b show all interactions between the combination of the four protein kinases through other proteins in second and third order respectively. From Figure 4a it is seen that there is about the same number of second order interactions for the four key protein kinases whereas the number of interactions vary more in the third order (see Figure 4b). It is interesting to note that in particular the Snf1 and Hog1 interacts extensively through third order interactions, whereas at the first level it is Snf1 and Pka1 that interacts most extensively. This indicated that Snf1 and PKA act in concert, i.e. on the same protein kinases, whereas Snf1 and Hog1 seem to integrate quite extensively through more complex routes.

The matrix representation of the protein-protein interaction network is interesting as it allows for easy analysis of interactions. It also allows for evaluation of the effects of deletion of specific proteins and how this affects different orders of interaction, and hence be used for easy evaluation of robustness of the network to different perturbations.

### Transcription Factors in the Reconstruction

**Figure 5:**
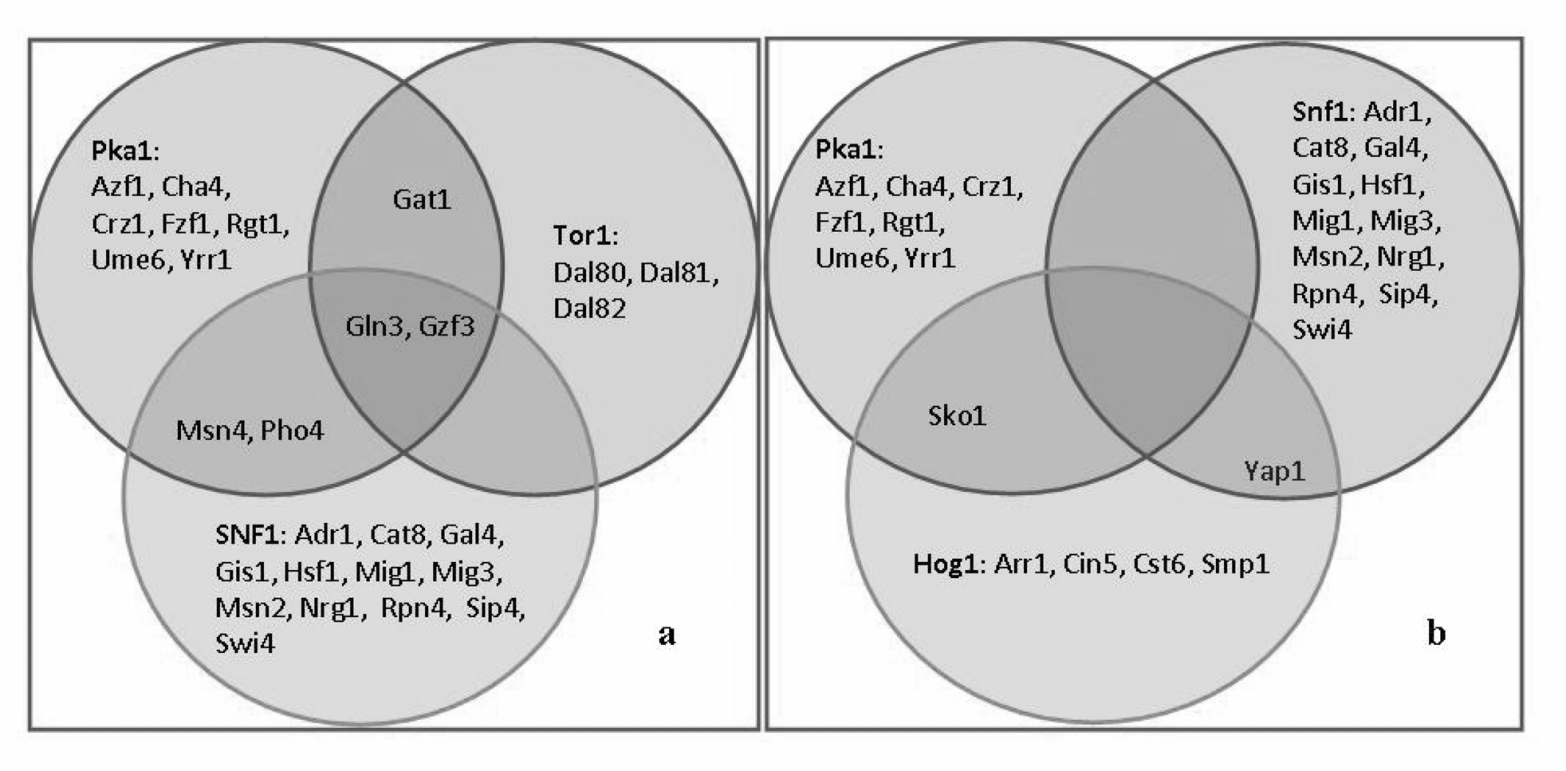
**Percentages of interactions of the key protein kinases with Transcription Factors in the protein-protein interaction map**. The transcription factor interactions with (a) Snf1, Tor1 and Pka1 and (b) Snf1, Hog1 and Pka1, respectively.

The reconstructed network contains a total of 44 proteins that have been annotated as transcription factors (TF). The transcription factor annotations were taken from http://www.yeastract.com[28]. The percentage of interactions with transcription factors were 41%, 32%, 13.5%, and 13.5% for Snf1, Pka1, Tor1 and Hog1, respectively. Five transcription factors were observed to interact with two of the hub proteins in the network and only two transcription factors had interactions with more than two of the hub proteins. Interactions of the transcription factors with the key protein kinases are visualized as Venn diagrams (Figure 5). The transcription factor interactions with Snf1, Tor1 and Pka1 and Snf1, Hog1 and Pka1 are shown in Figures 5a and 5b, respectively. The intercepts in the Venn diagrams show the shared transcription factor interactions among the three protein kinases. Hog1 and Tor1 share no common transcription factor interactions according to our current knowledge.

### Highly active sub-networks

**Figure 6:**
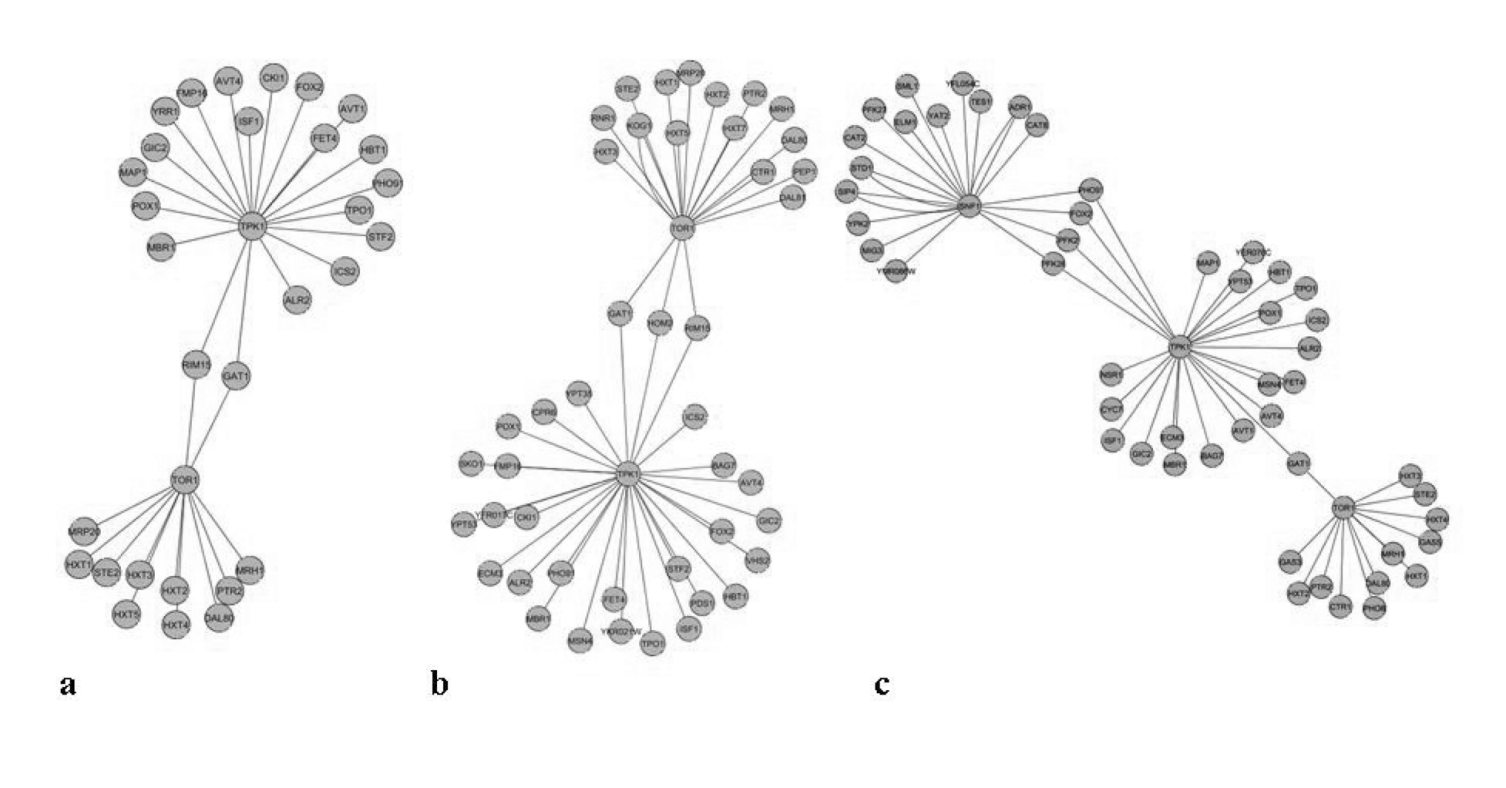
**Transcriptionally highly active sub-networks of the reconstructed protein-protein interaction network were identified under carbon- vs nitrogen- limited conditions at three different specific growth rates 0.2, 0.1 and 0.03 h^-1 ^for *S. cerevisiae***. The gene expression data were taken from Fazio *et al. *(2008) [6].

A search for transcriptionally highly active sub-networks [29,30] was performed in the reconstructed STP network to identify active transcriptional regulatory structures in *S. cerevisiae *in carbon- and nitrogen-limited conditions at three different specific growth rates: 0.20, 0.10 and 0.03 h^-1^. The search of highly active sub-networks was performed twice in series, since the first sub-networks identified were found to be large including a high fraction of nodes of the reconstructed network. The second search was performed inside the networks identified with the first search. The transcriptionally highly active subnetworks are shown in Figure 6. The first sub-networks included all the four hub proteins except in case of specific growth rate 0.10 h^-1 ^where Snf1 was not found to be included in the sub-network. The sub-networks from the second search included Tor1 and Pka1 interactions for growth rates of 0.10 h^-1 ^and 0.20 h^-1^. For the growth rate of 0.10 h^-1 ^the second sub-network showed that Tor1 and Pka1 had three common interaction proteins: Gat1, Hom2, and Rim15. These three proteins are annotated to have nitrogen metabolism or glucose repression dependent function. For the growth rate of 0.20 h^-1 ^the second sub-network showed that Tor1 and Pka1 shared interactions to Gat1 and Rim15 both related to nitrogen metabolism. For the lowest growth rate, 0.03 h^-1^, the second sub-network showed that Tor1 and Pka1 shared interactions only to Gat1 whereas Snf1 shared interactions to Fox2, Pfk2, Pfk26 and Pho91 with Pka1.

The proteins included in the highly active sub-networks having interactions with more than one key protein kinases are particularly interesting as they point to cross talk between the four different regulatory networks. Furthermore, the transcription factors having shared interactions to the protein kinases, that were present in the sub-networks, have probably been active regulators of transcription in the conditions studied. Thus, Tor1 and Pka1 share interactions to the transcription factors Gat1, Gln3 and Gzf3 that are involved in nitrogen metabolism and could have been mediators of the transcriptional changes in the studied conditions. Especially Gat1 is interesting as the directions of its interactions with the key kinases allow for information passing from Tor1 to Pka1. At the low specific growth rate (0.03 h^-1^) in addition to Tor1 and Pka1 also Snf1 interactions were found. Low specific growth rates have been proposed to cause stress in *S. cerevisiae *[28] and the Snf1 interaction network activity could be seen as a stress response. Thus, at the low specific growth rate there was observed a probable interaction between the global energy metabolism regulation and nutrient-sensing through common protein components. Tor1, Pka1 and Snf1 share interactions to the transcription factors Gln3 and Gzf3 that thus can be considered as probable active transcriptional regulators at low specific growth rate conditions.

### Comparison to published large scale protein-protein interaction networks

**Figure 7:**
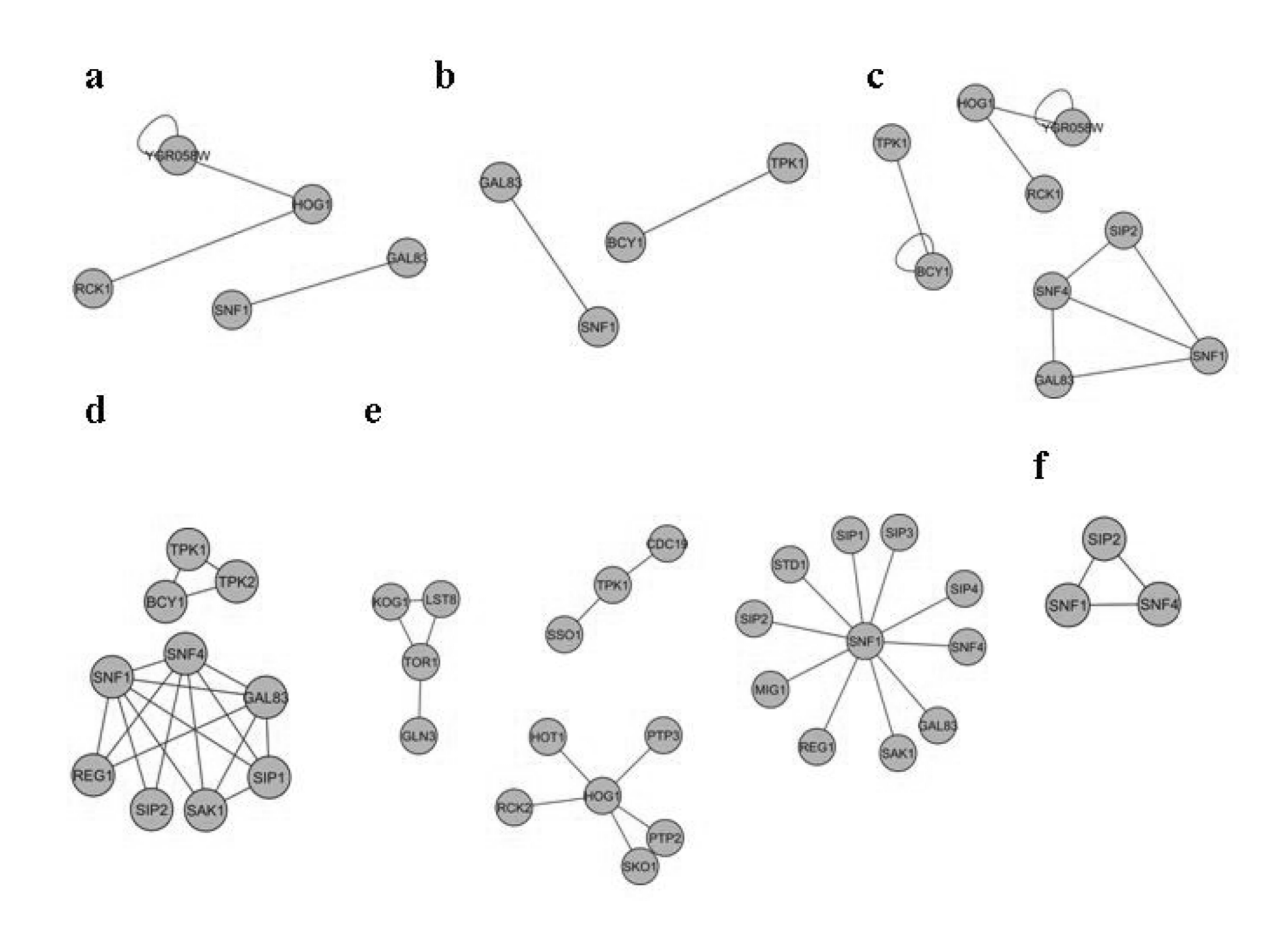
**Comparison of the published large scale protein-protein interaction networks: (a) Uetz-Screen, (b) Ito-core, (c) Y2H-Union, (d) Combined AP/MS, (e) LC-multiple, and (f) CCSB-YI1 for *S. cerevisiae *from Yu *et al. *2008 **[30].

The congruity of large scale protein-protein interaction networks and the large-scale study of the substrates of the yeast kinases by Snyder *et al. *(2005) [29] with the reconstructed protein-protein interaction network of the four key protein kinases studied here was investigated. The large scale protein-protein interaction networks investigated were Uetz-Screen [23], Ito-Core [24], Y2H-Union [30], Combined-AP/MS [31,32], LC-multiple [33,34] and CCSB-YI1 [30] all recently compared in extent and quality by Yu *et al*. (2008) [30]. Only a small fraction of the interactions to the four key protein kinases Snf1, Tor1, Hog1 and Pka1 were included in the previous large scale protein-protein interaction networks compared to what is reported here. None of the investigated large scale protein-protein interaction networks included proteins having interactions with more than one of the four key proteins. Thus, all the investigated large scale networks lacked the information on possible signal passing components between the regulatory systems around these four key protein kinases studied here (Figure 7).

The large-scale study of the substrates of protein kinases in yeast [29] included the substrates identified by proteome chip technology for Snf1 and Pka1. In this study Snf1 and Pka1 was found to share 55 substrates *in vitro *of which 53 proteins with well annotated interactions to both Snf1 and Pka1 are also included in the network reconstruction presented here. The other two key protein kinases Tor1 and Hog1 were not included in the study by Snyder *et al. *(2005) [29] and thus it lacked the information on the possible signal transfer components between the pathways of all the four key protein kinases.

## Discussion

As a starting point for the development of a comprehensive protein-protein interaction network spanning four key protein kinases, we performed a manual annotation process that combines different kinds of information for every single protein and interactions. The results are presented as files in SBML format (.xml) available on our website http://www.sysbio.se/users/Subir. This SBML format .xml file will open directly in CellDesigner and the full interaction map describes all the available information. The information of protein-protein interactions for these four key protein kinases represents an extensive starting point for further reconstruction of protein-protein interactions in yeast. The main contribution of this paper is the collection of all the interaction partners of the four key protein kinases from different sources into a protein-protein interaction network and to describe the full annotation of the interactions. The reconstructed network serves as an initial platform for reconstruction and annotation of a genome-wide signal transduction network. The presented network includes the four key protein kinases of nutrient sensing and regulation of metabolism, their interaction partners and all the interactions between the key protein kinases and the other proteins. Thus, the topology of the network is predetermined.

**Figure 8:**
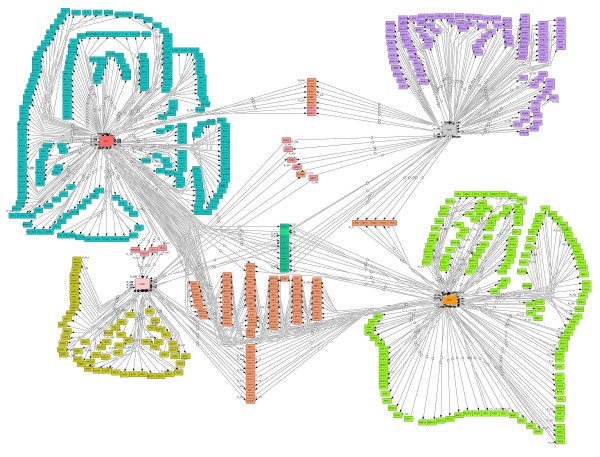
**Illustration of the global protein-protein interaction network involving the four key protein kinases Snf1, Hog1, Tor1 and Pka1**.

Our reconstruction process resulted in a network that consists of 623 proteins and 779 protein-protein interactions. In line with the nomenclature used for genome-scale metabolic models we propose to call this reconstructed network ppSK623 (see Figure 8). In this paper, interactions are fully annotated in the reconstruction which gives more than just a score for a user to evaluate how reliable the interaction is in his/her case and can be able to score the different sources that have been used to collect the interactions (see additional file 1 for the cell designer file with all protein list in .xls form).

Furthermore, compared to different databases like STRING where only 185 similar interactions have scores our database covered 594 more interactions than reported in STRING based only on the four key protein kinases. Annotated information on the interactions is given in the CellDesigner file that includes all information together. Furthermore the interactions between Transcription Factors and different protein kinases provides a scaffold for building more detailed models, e.g. to study the dynamics of signal transduction pathways.

The analysis of transcriptionally highly active sub-networks of the integrated protein-protein interaction reconstruction allowed for identification of possible information carriers between the interaction networks of the four key protein kinases in *S. cerevisiae *in carbon- vs nitrogen-limited conditions at three different specific growth rates. This would not have been feasible using separate regulatory networks for the kinases.

None of the studied large protein-protein interaction networks included the wealth of information on the probable global metabolic regulation and interactions between the nutrient-sensing protein kinases included in the reconstructed network described in this study. Thus, the large protein-protein interaction networks could not have supported the above discussed analysis of integrated response of the pathways and identification of probable information carriers between signal transduction pathways of the four key protein kinases. This is the first attempt to combine the signal transduction networks of the four key protein kinases. Previously the interaction networks of the key protein kinases have been studied separately. The reconstructed protein-protein interaction network serves as a framework for analysis of nutrient sensing and global regulation of metabolism, for analysis of data, for analysis of information transfer between the regulatory networks of the individual protein kinases and as an initial platform where the reconstruction and annotation of global signal transduction network can be conveniently continued. The modelling format is carefully chosen to suit continuation of the reconstruction and annotation.

## Conclusions

The SBML-encoded version representation of the model is made available in one of the preferred software platforms for system biology, namely CellDesigner. We have examined the SBML format in .XML state in CellDesigner and shown that it loads successfully for visualization.

Subsets of this model are relevant for some applications and through the CellDesigner representation the data are made available as a database that facilitates easy searching in the network. An important function of the CellDesigner format is that this is an open ended source that allows each researcher to edit the model and hereby improve the model based on new information. Our reconstruction and the used platform are, therefore, well suited for initiating a community effort towards reconstruction of a highly annotated protein-protein interaction network for yeast. This nutrient sensing and global metabolic regulation map in yeast will offer as a valuable resource for the research of S. cerevisiae and also provides insight into this important regulatory network in eukaryotes in general.

## Methods

Annotation of the key STP of *S. cerevisiae *has made it possible to obtain information of physical interaction between the proteins involved in this reconstruction. Information on this is available in the *Saccharomyces *Genome Database (SGD) that includes much information for individual proteins, but little information about protein networks. In our study we combine all the proteins involved in key nutrient sensing pathway, i.e. pathways involving the protein kinases Snf1, Tor1, Hog1 and Pka1. To provide a platform that allows for future revisions and expansion of the interaction network we organized the information about all the proteins and their interaction information in CellDesigner, which allows easy transfer also to Cytoscape.

### Reconstruction of protein-protein interaction network

Figure 1 demonstrates the reconstruction process. In brief, we integrated all the different types of available information to present and annotate a single protein-protein interaction network using different sources like databases, textbooks, publications and reviews. In this protein-protein interaction map all proteins and interactions are referenced, both in terms of proteins and interactions.

### Network modeling in CellDesigner and Cytoscape

The network reconstruction was built in the CellDesigner network modeling software and both CellDesigner and Cytoscape were utilized for visualization of the network. The network nodes and edges were annotated in CellDesigner, i.e. there is a reference for each protein and its interactions. The advantage of CellDesigner is that a lot of information can be stored in the notes part for each protein and interaction. The network model and the annotation data are stored in the same .xml file in CellDesigner. The reconstruction interaction network can be exported to Cytoscape, and both software platforms have a number of adjustable visualization options.

### Databases

Many different databases are available for *S. cerevisiae*. Here we used BIOGRID, SGD and specific research papers as information input to the annotation process. The biological general repository [35] for interaction datasets (BIOGRID) is one of the most important datasets for protein-protein interaction but the *Saccharomyces *Genome Database (SGD) is also providing much information. Our reconstruction model combines information from these different databases to make a common platform to study the interaction between the studied protein kinases. SGD and BIOGRID collect and organize biological information on proteins and their interactions of the budding yeast *S. cerevisiae*, but we also used published data [4] from traditional experimental methods and also from computational predictions, as these can give additional valuable information.

### Identification of highly active sub-networks

We identified highly active sub-networks in the reconstructed protein-protein interaction network for differential gene expression of *S. cerevisiae *in carbon- and nitrogen-limited conditions at three different specific growth rates. The Affymetrix gene expression data were taken from Fazio *et al. *(2008) [6]. The algorithm used for identification of highly active sub-networks was the one developed by Patil *et al. *(2005) [36] based on the original work by Ideker *et al. *(2002) [37]. The analysis of Affymetrix microarray data was done using R/Bioconductor, version 2.6.1 [38]. The raw data were normalised with Robust Multichip Average (RMA) normalisation [39-41]. Statistical differences in expression were calculated using linear modelling tools of the *limma *package [42,43]. For each gene, a linear model was fitted by the least squares method and differential expression within pairs of experimental conditions was computed using the empirical Bayesian approach [44,45]. Empirical Bayes adjusted p-values of statistical significance of expression changes were used to score the nodes in the network for identification of transcriptionally highly active sub-networks in the reconstructed network of the key STP in *S. cerevisiae*. The algorithm developed by Patil *et al. *(2005) [36] converted the p-values to Z scores of the protein nodes by using the inverse normal cumulative distribution (θ-1), calculated then a combined score Zs of the protein node Z scores for a connected subnetwork s, and corrected the Zs score for the background distribution. Then the algorithm utilizes simulated annealing for solving the problem of finding the subnetwork having the highest score.

## Authors' contributions

SKN created and tested the key four protein kinase interactions in CellDesigner and wrote the manuscript. PJ provided input into the analysis and were involved in writing the manuscript. JN supervised the work and were involved in writing the manuscript. All authors read and approved the final version of the manuscript.

## Supplementary Material

Additional file 1 **Detail demonstration of the proteins in PPI (protein protein interaction)**. The file contains a list of all proteins used in this study visualized in Cell Designer from SBML file and also explained the protein list in .xls form. The binary matrix of the proteins used in this study is also included. In addition, the Renata Usaite paper is also added for more information. Click here for file
