# Supplementary material for: Reconstruction of the yeast protein-protein interaction network involved in nutrient sensing and global metabolic regulation
Source: BMC Syst Biol. 2010 May 25;4:68. doi: 10.1186/1752-0509-4-68 (PMC2889877; doi:10.1186/1752-0509-4-68)
Supplement: Additional file 1 — Detail demonstration of the proteins in PPI (protein protein interaction). The file contains a list of all proteins used in this study visualized in Cell Designer from SBML file and also explained the protein list in .xls form. The binary matrix of the proteins used in this study is also included. In addition, the Renata Usaite paper is also added for more information. [file 1752-0509-4-68-S1.DOC]

**Supplementary information**

**Reconstruction of the yeast protein-protein interaction network involved in nutrient sensing and global metabolic regulation**

**Subir Kumar Nandy, Paula Jouhten and Jens Nielsen***

*Corresponding Author:

Prof. Jens Nielsen,

Systems Biology Group

Department of Chemical and Biological Engineering

Chalmers University of Technology

Kemivägen 10, SE-412 96 Gothenburg, Sweden.

Email:     [nielsenj@chalmers.se](mailto:nielsenj@chalmers.se)

Tel:      +46-31 772 38 04.

Fax:        +46 31 772 38 01.

**Abstract**

Here we describe an annotated reconstruction of four key nutrient-sensing and metabolic regulatory signal transduction pathways (STP) of the model organism *Saccharomyces cerevisiae*. The reconstructed STP network includes a full protein-protein interaction network including the key nodes Snf1, Tor1, Hog1 and Pka1.  The network includes a total of 623 structural open reading frames (ORFs) and 779 protein-protein interactions. A number of proteins were identified having interactions with more than one of the protein kinases. The fully reconstructed interaction network includes all the information available in separate databases for all the proteins included in the network (nodes) and for all the interactions between them (edges). The annotated information is readily available utilizing the functionalities of network modelling tools such as Cytoscape and Cell Designer.

The reported fully annotated interaction model serves as a platform for integrated systems biology studies of nutrient sensing and regulation in *S. cerevisiae*. Furthermore, we propose this annotated reconstruction as a first step towards generation of an extensive annotated protein-protein interaction network of signal transduction and metabolic regulation in this yeast.

**Supplementary Data**

**SBML file of full annotation of four key protein kinases** :: [Download file](http://www.sysbio.se/supp/reconstructionofPPI/material/Full interaction Snf1 Tor1 Pka1 Hog1 with nodes and edges.xml)

**Protein interaction list.xlsx** : [Download file](http://www.sysbio.se/supp/reconstructionofPPI/material/Protein Interaction list.xlsx).xls: [Download file](http://www.sysbio.se/supp/reconstructionofPPI/material/Protein Interaction list.xls)

**Protein list.xlsx** : [Download file](http://www.sysbio.se/supp/reconstructionofPPI/material/Protein list.xlsx).xls: [Download file](http://www.sysbio.se/supp/reconstructionofPPI/material/Protein list.xls)

**Binary matrix .xlsx** : [Download file](http://www.sysbio.se/supp/reconstructionofPPI/material/BINARY MATRIX.xlsx).xls: [Download file](http://www.sysbio.se/supp/reconstructionofPPI/material/BINARY MATRIX.xls)

Reference 4: Renata Usaite paper .pdf: [Download file](http://www.sysbio.se/supp/reconstructionofPPI/material/Renata Usaite.pdf)
